# A wearable eddy current based pulmonary function sensor for continuous non-contact point-of-care monitoring during the COVID-19 pandemic

**DOI:** 10.1038/s41598-021-99682-2

**Published:** 2021-10-11

**Authors:** Shane Shahrestani, Tzu-Chieh Chou, Kuang-Ming Shang, Gabriel Zada, Zea Borok, Adupa P. Rao, Yu-Chong Tai

**Affiliations:** 1 Department of Medical Engineering, California Institute of Technology, 1200 E California Blvd, MC 136-93, Pasadena, CA 91125 USA; 2 Department of Electrical Engineering, California Institute of Technology, Pasadena, CA USA; 3 Department of Neurosurgery, Keck School of Medicine, University of Southern California, Los Angeles, CA USA; 4 Division of Pulmonary, Critical Care and Sleep Medicine, Department of Medicine, Keck School of Medicine, University of Southern California, Los Angeles, CA USA

**Keywords:** Translational research, Respiratory distress syndrome, Biomedical engineering

## Abstract

Pulmonary function testing (PFT) allows for quantitative analysis of lung function. However, as a result of the coronavirus disease 2019 (COVID-19) pandemic, a majority of international medical societies have postponed PFTs in an effort to mitigate disease transmission, complicating the continuity of care in high-risk patients diagnosed with COVID-19 or preexisting lung pathologies. Here, we describe the development of a non-contact wearable pulmonary sensor for pulmonary waveform analysis, pulmonary volume quantification, and crude thoracic imaging using the eddy current (EC) phenomenon. Statistical regression analysis is performed to confirm the predictive validity of the sensor, and all data are continuously and digitally stored with a sampling rate of 6,660 samples/second. Wearable pulmonary function sensors may facilitate rapid point-of-care monitoring for high-risk individuals, especially during the COVID-19 pandemic, and easily interface with patient hospital records or telehealth services.

## Introduction

Pulmonary function testing (PFT) involves comprehensive evaluation of the lungs to provide objective and quantifiable metrics for pulmonary function. Oftentimes, PFTs are indicated when the clinician suspects obstructive lung disease, such as chronic obstructive pulmonary disease (COPD) and asthma, or restrictive lung disease, such as pulmonary fibrosis or sarcoidosis. In contemporary clinical practice, PFTs are most commonly performed using spirometry, and occasionally with lung plethysmography^1–3^. However, the American Thoracic Society and many comparable international societies have recommended postponing PFTs during the coronavirus disease 2019 (COVID-19) pandemic, due to the high risk of COVID-19 transmission during the outpatient testing visit^4–8^. While these restrictions were put in place through expert guidance to prevent the spread of disease, they have made it difficult to gauge pulmonary function in patients with previously diagnosed lung disease as well as those recovering from COVID-19 pneumonia whom require close follow-up examination.

As such, wearable continuous pulmonary biosensors may play a unique role in evaluating pulmonary function without requiring direct inpatient or outpatient examination. In fact, several pulmonary biosensors have been previously described in the literature. Devices utilizing accelerometers have been shown to accurately characterize the respiratory waveform through anteroposterior displacement of the thorax during breathing^9^. Other groups have developed acoustic-based sensors that monitor air movement near the nose, or air moving through an airway, to monitor respiration^10,11^. Similarly, mechanical strain sensors, placed on the chest like a piece of tape, have been implemented by some groups to accurately measure the respiratory waveform in addition to approximation of non-statistically significant ratios of forced expiratory volume in one second (FEV_1_) and forced vital capacity (FVC)^12^. However, many of these sensors are unable to accurately gauge respiration volume and relevant pulmonary metrics, and strain-based sensors require calibration and tight contact with the patient’s skin to yield accurate results^12^.

With current limitations in mind, our group describes the development of a non-contact, wearable, and continuous pulmonary function sensor capable of detecting both respiratory waveforms and volumes. The sensor relies on the generation of microtesla-level magnetic fields and the eddy current (EC) phenomenon to track changes in conductivity during the respiratory cycle. We demonstrate that our wearable electromagnetic sensor is safe and can continuously measure relevant pulmonary function with a high degree of accuracy through clothing. Additionally, implementation of conductivity-based scanning allowed for crude imaging of the thoracic cavity due to the wide range of conductivities of organs contained within. The development of such a device may allow for efficient non-contact PFTs during the COVID-19 pandemic, provide relevant respiratory metrics for remote clinical assessment, and monitor conductivity changes within the lungs resulting from edematous or inflammatory lung pathologies.

## Methods

### Sensor development

In our experiment, we constructed a solenoid coil with an outer diameter of 11.4 cm and 6 turns out of 46 AWG Litz wire. These parameters were selected after building several coils and determining appropriate scanning depths for both static and dynamic experiments. The coil was wound around a non-conductive plastic scaffold and connected to the Texas Instruments LDC 1101 inductance-to-digital converting chip^13^. Non-destructive techniques based on the EC phenomenon are widely used to test the presence, quantity, and integrity of various conductive materials, most commonly metals^14^. Eddy currents generated by a conductive material flow in the direction that decreases coil inductance and coil parallel resistance (R_p_), which may be used to quantify the degree of EC produced^15,16^. As a result, this change in resistance then becomes proportional to the conductivity of the material placed in front of the sensor.

The sensor was designed to have a resonant frequency at 1 megahertz (MHz), which was confirmed through analysis with the LDC 1101 graphical user interface (GUI). Several factors were considered when choosing 1 MHz. First, the LDC 1101 inductance-to-digital converter chip only operates at frequencies between 500 kHz and 10 MHz. Furthermore, we know that higher frequencies have shorter penetration into tissues and lower frequencies have higher penetration into tissues. After much thought, we settled on building the coil to operate at a frequency of 1 MHz to balance the pros and cons of sensor circuit limitations, available conductivity measurements from the literature, and penetration depth into the chest. In order to minimize capacitive coupling and reduce the noise of our sensor, we covered the lateral sensor border with thin ferrite sheets, leaving the end facing the target unshielded. The use of magnetic shielding with ferrite material helped to redirect the magnetic field distribution toward the target and block the electromagnetic interference from external sources. The average relative permeability of the ferrite sheet (MULL12060-000, Laird Technologies Inc.) is approximately 135 from 1 to 10 MHz, which encapsulates the frequency range used in this study^17^. Prior to testing, the sensor sampling rate was found to be 6,660 samples/second and the signal-to-noise ratio of the sensor was found to be 9.4. Characterization of the sensor magnetic field using both Biot-Savart’s law (0.1786μT) and multiphysics modeling (0.1μT) confirmed a magnitude of several microteslas.

### Signal processing

All data was converted into digital signals using the inductance-to-digital converting chip and stored on a local laptop for analysis in MATLAB. Savitzky-Golay and simple Gaussian filters from the Signal Processing Toolbox were implemented to smooth the respiratory time series waveforms and to remove high frequency noise. Local maxima and minima were located within the respiratory diagrams and the difference between the peaks and troughs were averaged for each individual trial, yielding the average change in R_p_ per breath. The total number of breathing cycles within the data, defined as one inhalation paired with one exhalation, as well as the scanning time were utilized to predict the respiratory rate of volunteers according to the following equation: 60 s/Scanning Time (seconds)*Number of Cycles.

### Ethics

Institutional Review Board (IRB) approval was obtained at the California Institute of Technology (IRB Number: 20–1005) for non-randomized human feasibility testing of the respiratory sensor. All methods were performed in accordance with the relevant guidelines and regulations.

### Human testing

All participants tested negative for COVID-19 before data collection, and patients with any pulmonary pathology were ineligible for recruitment. Informed consent was obtained from each study participant and scanning data was obtained by S.S. A total of 4 participants were recruited (three participants tested three times). Each participant was asked to lay prone on a non-conductive surface while 8 vertical scans were obtained across the chest. In addition, the patient's chest was recorded, with the sensor placed on the mid-sternum, while sitting straight up. In all trials, participants were instructed to breathe through their nose with regular, non-labored respiratory rhythms. It was ensured that each patient was not wearing any metallic items (jewelry, clothing, etc.) and that the immediate surrounding was void of any metallic items to minimize interference with the electromagnetic sensor.

### Pulmonary function testing

The Medical International Research (MIR) Spirobank II® spirometer was utilized to record baseline PFTs for each volunteer in the study according to instructions of use. Spirometry was recorded immediately following scanning with the sensor. This device was utilized to obtain the FEV_1_ and FVC of each participant to compare with the results obtained with the EC sensor described in this study.

### Heatmap processing

The anterior chest was scanned using 8 equidistant vertical scanning paths, starting at the left midaxillary line to the right midaxillary line. The length of each scan spanned from the lower rib border up to the clavicles/sternal notch. Data from the sensors was filtered to remove high frequency noise. The data collected from each row was down-sampled from > 50,000 data points collected during scanning to eight averaged points. We use the eight data points from the eight scanning rows to create an 8 × 8 interpolated conductivity heatmap of the chest, with brighter areas indicating higher probability of more conductive organs or regions and darker areas indicating a higher probability of air or non-conductive tissue.

### Statistical analysis

Basic demographics and relevant values were visualized using GraphPad Prism 8. Statistical analysis was performed in MATLAB and R and aimed to establish the accuracy of sensor resistance values in predicting pulmonary volume changes. Specifically, regression analyses were performed to investigate the correlation between spirometry, respiratory rates, and EC sensor values. The equation for these regression lines was also used to calculate the predicted FEV, FVC_1_, and RR values. Bland–Altman plots were created to gauge agreement between actual and predicted pulmonary metrics. Both *p* values and R-squared values were reported for each regression model to evaluate goodness-of fit. *P* values < 0.05 were considered statistically significant.

## Results

### Operating principles

**Figure 1:**
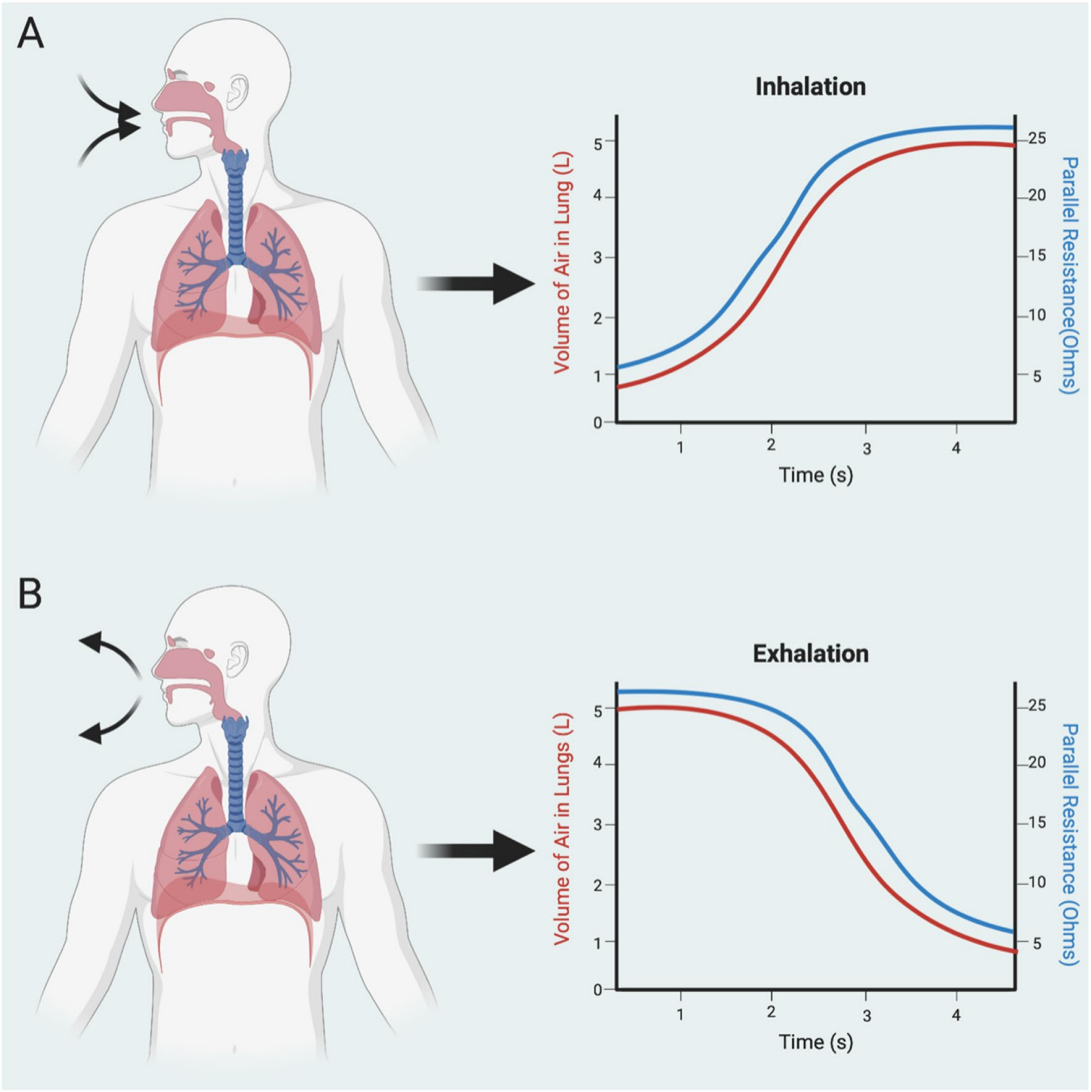
Correlation between resistance and lung air volume. (**A**) As the volume of air in the lungs increases during inhalation, so do the parallel resistance values queried by the sensor. This is because room air is non-conductive, resulting in reduced eddy currents and thus, increased parallel resistance. (**B**) Conversely, exhalation decreases the volume of air within the thoracic cavity, reducing lung air volume and parallel resistance.

Chest movement produced with inspiration and expiration displaced the sensor in the anteroposterior direction with the chest wall, with each inhaled breath replacing conductive tissue and vasculature within the sensor magnetic field space with non-conductive room air. Eddy currents generated due to the decrease in local conductivity during inhalation resulted in increased R_p_ measurements taken by the chip (Fig. 1A & Supplementary Fig. 4). Upon exhalation, non-conductive air in the lungs is released and the corresponding reduction in chest diameter brings conductive tissue back into the range of the sensor, thus increasing eddy currents and decreasing R_p_ measurements (Fig. 1B).

**Figure 2:**
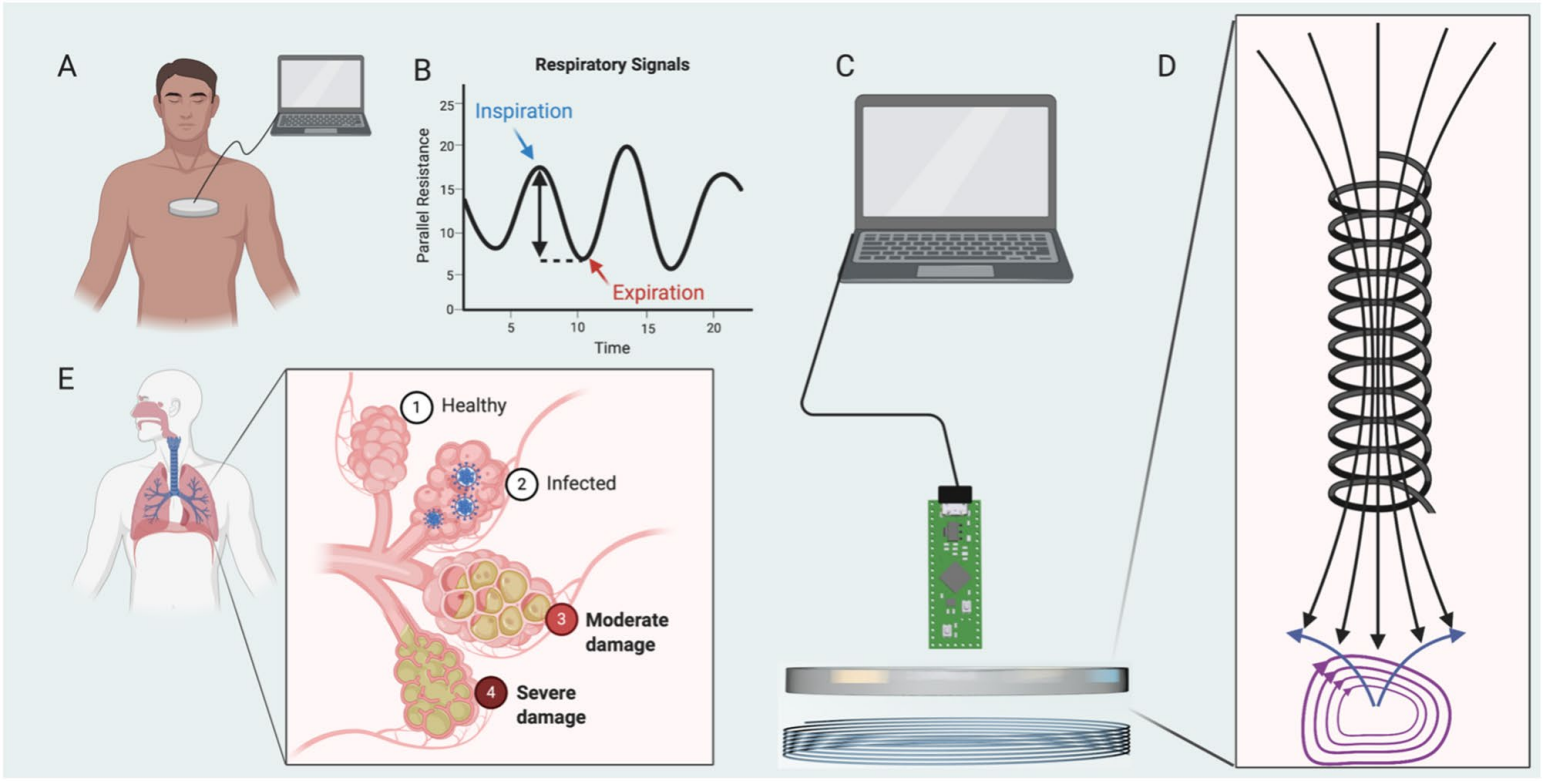
Experimental setup and sensor principles. (**A**) During static pulmonary testing, the sensor is placed on the mid-chest, with data recorded and stored on a local computer. (**B**) Fluctuations in parallel resistance during scanning result in a cyclical time-series waveform, with inspiration resulting in local maxima and expiration resulting in local minima. The difference between maxima and minima yield parallel resistance changes per breath. (**C**) The sensor is constructed as a solenoid with 6 turns on a plastic scaffold connected to a commercial inductance-to-digital converting chip which is powered via a USB connection. (**D**) The magnetic field generated by the solenoid induced eddy currents within the target proportional to target conductivity, which produce counteracting magnetic fields that influence coil resistance. € Alveolar damage, following COVID-19 for example, presents with varying degrees of damage which may present differently. Early-stage pulmonary dysfunction may be queried with such a sensor.

In contrast to similar biosensors that require extensive calibration or tight contact with the individual to produce accurate results, our pulmonary sensor generated pulmonary waveforms through the shirt after being freely placed on the chest of participants in a non-contact manner (Fig. 2A). As such, the anteroposterior motion of the chest during the breathing cycle results in a cyclical change in coil R_p_, which is converted to digital signals and stored on a local computer for analysis.

Through signal processing and smoothing, it is then possible to generate an approximate respiratory waveform using the sensor. By subtracting the lower R_p_ resulting from expiration from the higher R_p_ resulting from inspiration, it is possible to approximate the amount of signal generated from each breath (Fig. 2B). This change in resistance is directly proportional to the amount of EC generated or lost with each exhalation or inhalation, respectively. Changes in R_p_ for each breath may then be averaged across the respiratory time-series of participants to yield the mean change in R_p_ per breath.

The sensor architecture developed for this application consists of a sensor coil paired with a capacitor to form an electrical resonant circuit, which is comparable to the architecture of previous EC sensors used in industry for distance gauging consisting of a bridge circuit that measures the sensor coil impedance^14^. A schematic for this sensor is shown in Fig. 2C, and the sensor components, chest placement, and study setup are illustrated in Supplemental Fig. 1.

When the sensor is brought in close proximity with a conductive material, such as the heart, aorta, liver, and vessels of the chest, the magnetic field generated within the solenoid coil will generate eddy currents in the target, which will produce a counteracting magnetic field resulting in a decrease in coil inductance and R_p_ (Fig. 2D). The magnitude of counteracting magnetic fields is directly proportional to the conductivity of the target material and the orthogonal distance between the sensor and the target^18^. Because lung pathology is characterized as either obstructive or restrictive, changes in the pulmonary time-series waveform generated by such a sensor may provide clinically relevant patterns aiding in diagnosis following alveolar damage (Fig. 2E). Continuous monitoring over long periods of time may be able to detect slow progression of lung disease using non-contact methods, which is particularly of interest for specific lung pathologies including pulmonary fibrosis and COVID-19 acute respiratory distress syndrome (ARDS).

### Static scanning

To determine the respiratory waveform and respiratory rate (RR) of participants, 15–25 s worth of data was recorded from the sensor while it was placed directly on the sternum (Fig. 2A). Other scanning sites on the chest were investigated, but placement on the sternum generated the greatest and most consistent signal, while allowing for data collection from both lung fields simultaneously. Signals generated by respiration were collected in real-time, and post-collection data processing was implemented to visualize smooth transitions between inhalation and exhalation.

**Figure 3:**
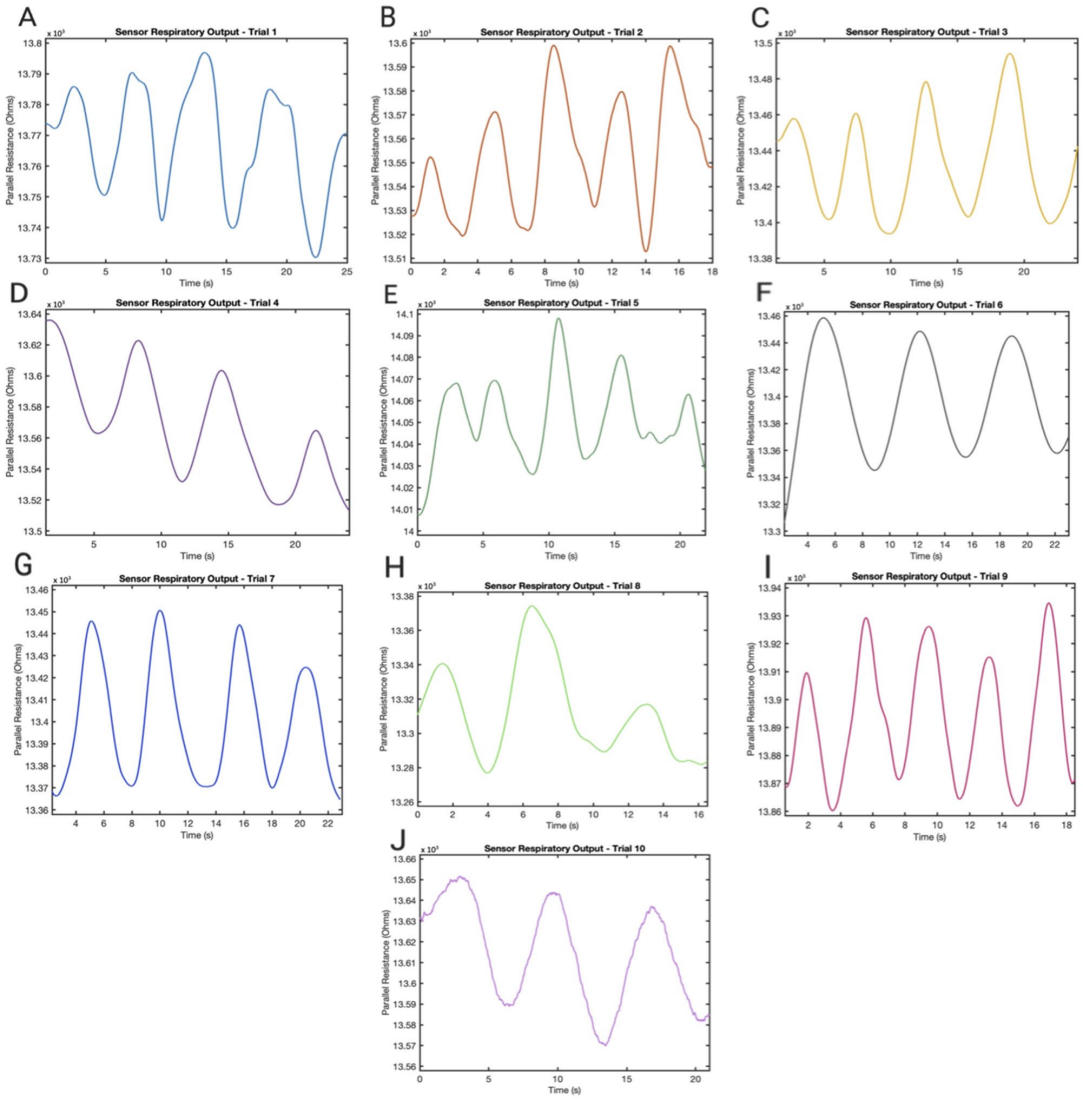
Sensor respiratory waveform output. (**A-J**) Each waveform shows the unique pulmonary waveform, as generated by the sensor, for each trial in this study.

**Figure 4:**
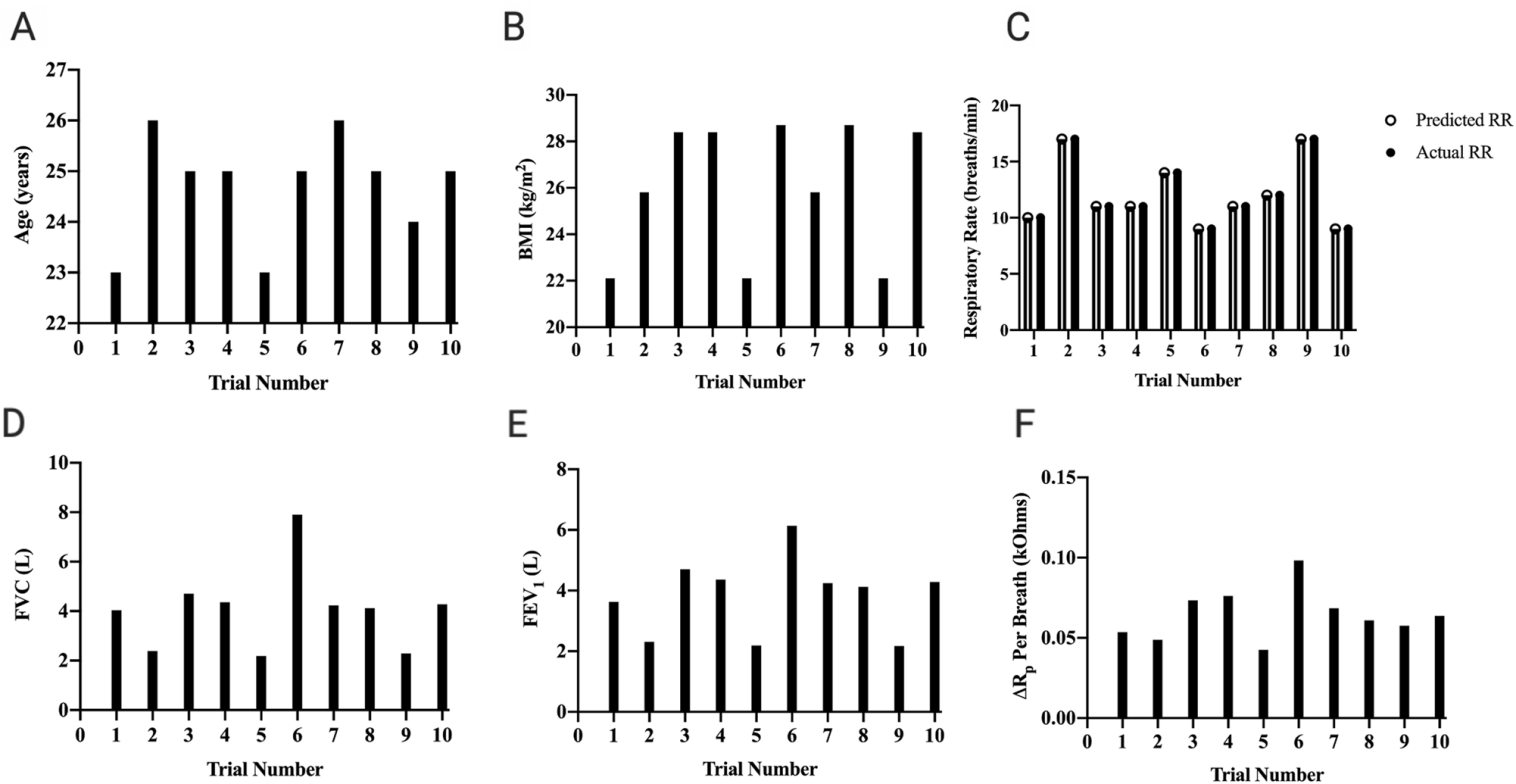
Participant variables and demographics. (**A**) Age of participants by trial. (**B**) Body mass index (BMI) of participants by trial. (**C**) Respiratory rate of participants by trial. (**D**) Forced vital capacity (FVC) of participants by trial. (**E**) Forced expiratory volume in one second (FEV_1_) of participants by trial. (**F**) Change in R_p_ of participants by trial.

Pulmonary waveforms following human testing were generated for each trial (Fig. 3A–J) (Supplementary Fig. 2). Between all volunteers, the approximate R_p_ range for respiratory cycles ranged between a minimum of 13.2 kOhms and a maximum of 14.1 kOhms. For most samples, basic data smoothing resulted in periodic waveforms, while others showed minimal amounts of hysteresis or sensor drift over time. However, much of the hysteresis/drift was seen when recording for more than 10 min rather than several seconds, and future implementation of continuous PFTs using this device should be performed for several minutes at a time, rather than continuously leaving the sensor on for hours or days. This is a limitation of the commercial inductance-to-digital converter and can eventually be overcome with a more sophisticated circuit. Basic demographics for all participants were recorded, including age and body mass index (BMI) (Fig. 4A,B). The predicted RR was also calculated using the time-series of each trial and compared to the actual participant RRs (Fig. 4C). As previously described, differences were taken between local maxima and minima in the sensor output time-series waveforms to estimate the mean change in R_p_ breath (Fig. 4F) per trial.

Immediately following scanning with our sensor, PFTs were recorded using the MIR Spirobank II®, which is a commercially available spirometer. Previous studies have shown that the Spirobank II® is an accurate and appropriate research tool for pulmonary testing^19^. Through this step, the FEV_1_ and FVC of each participant was queried for comparison with R_p_ changes seen using the sensor (Fig. 4D,E). FEV_1_ to FVC ratios less than 80% have been highly correlated with obstructive lung disease due to a greater decrease in FEV_1_ than FVC^20,21^. As such, it was ensured that the ratio of FEV_1_ to FVC was > 80% for all patients, with a mean of 95.9% in the cohort.

**Figure 5:**
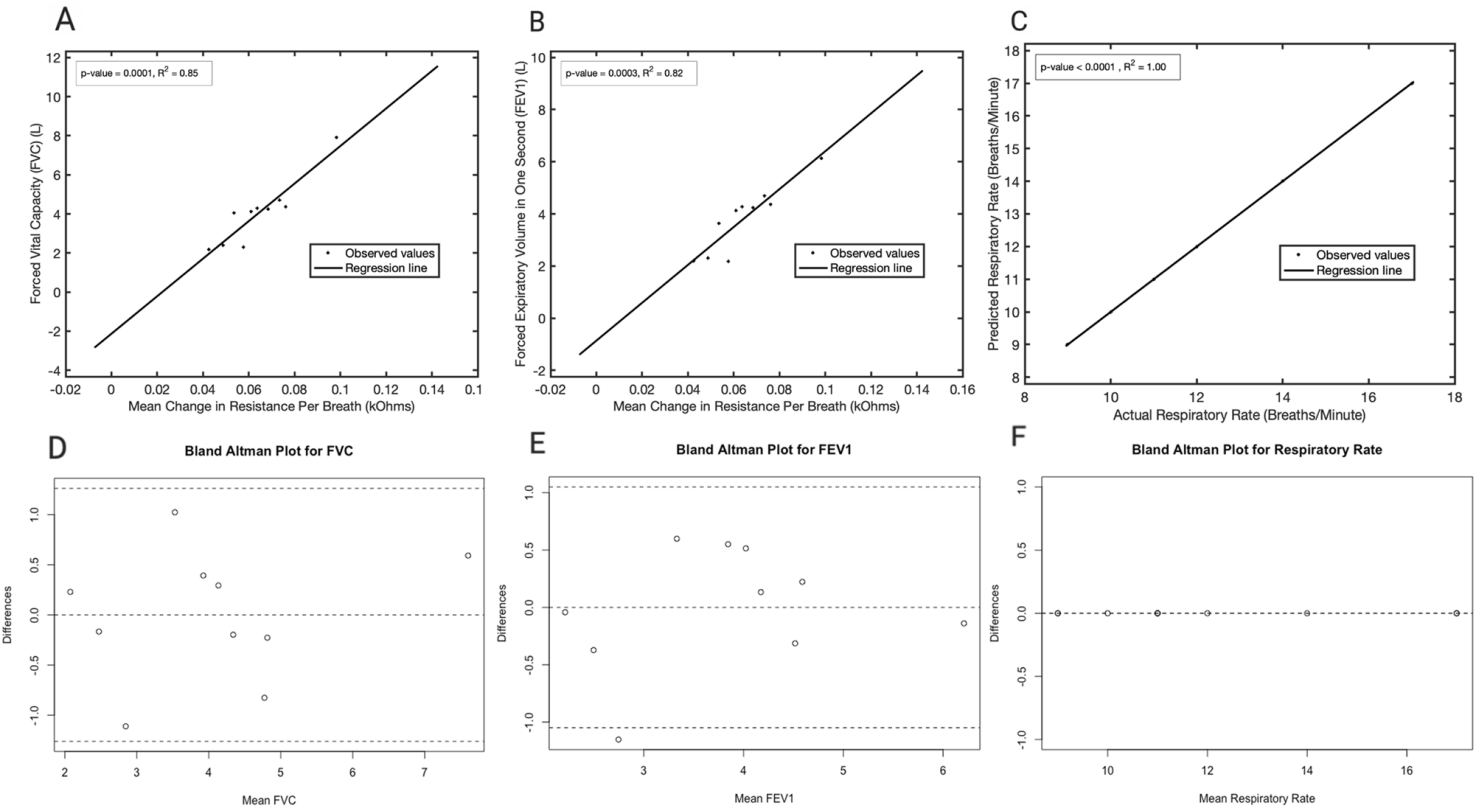
Regression analyses. (**A**) Linear regression analysis correlating mean change in parallel resistance per breath and FVC. (**B**) Linear regression analysis correlating mean change in parallel resistance per breath and FEV_1_. (**C**) Linear regression analysis correlating predicted and actual respiratory rates. (**D–F**) Bland–Altman plots for FVC, FEV_1_, and RR respectively.

Statistical regression analysis was performed to demonstrate the accuracy of sensor output as a predictor for RR, FEV_1_, and FVC. Correlation between mean change in R_p_ per breath and all three pulmonary variables demonstrated a statistically significant positive correlation, with RR showing the greatest predictive value (R^2^ = 1.00, *p* value < 0.0001; Fig. 5C). With respect to pulmonary volume metrics, regression analysis demonstrated that mean change in R_p_ per breath was more closely correlated with FEV_1_ (R^2^ = 0.82, *p* value = 0.0003; Fig. 5B) than FVC (R^2^ = 0.85, *p* value = 0.0001; Fig. 5A). Equations were developed for each regression line as follows: y = 96.006x − 2.1289 for FVC; y = 72.662x − 0.8633 for FEV_1_; and y = x for RR. Bland–Altman testing demonstrated acceptable agreement between the actual and regression-predicted values for FVC, FEV_1_, and RR (Fig. 5D-F).

### Dynamic scanning

**Figure 6:**
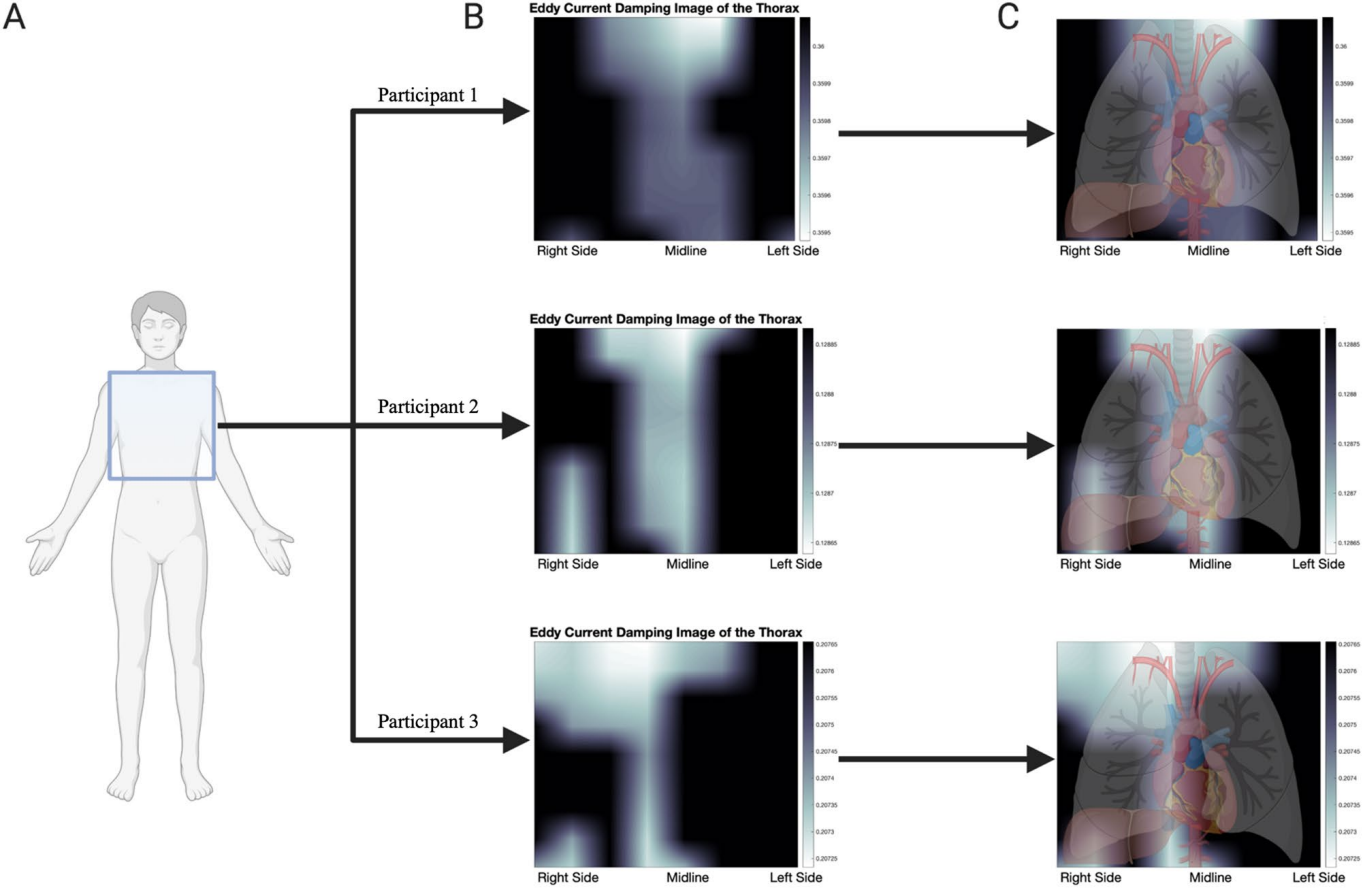
Heatmap images and finite element modeling. (**A**) Eight vertical scanning paths were made around the chest. (**B**) Heatmaps generated using R_p_ values during dynamic scanning of the chest for three different study participants. More conductive regions are shown brighter (higher eddy currents, lower R_p_), while less conductive regions are shown darker. (**C**) Heatmaps of the chest with overlaid anatomical landmarks demonstrating high conductivity of vasculature, heart, and liver for three different study participants.

To achieve preliminary conductivity-based imaging of the thoracic cavity, the sensor was scanned vertically across 8 equidistant rows on the chest. Scanning the anterior chest wall started at the left mid-axillary line (row 1) and continued to the right mid-axillary line (row 8), vertically from the inferior border of the ribs to the clavicles superiorly (Supplementary Fig. 3). Real-time data was collected during each chest scan (Fig. 6A) and data from all 8 scans were normalized, smoothed, and plotted as individual heatmaps for three participants (Fig. 6B).

According to the underlying physics of the sensor, more conductive regions will result in lower R_p_ values while less conductive regions will result in higher R_p_ values. Within the thoracic cavity, large vessels branching off of the aorta and the heart would be expected to generate the largest EC changes, while the air-filled lungs would produce the smallest EC. On the same note, previous studies have found that the liver has the same conductivity as the heart at 1 MHz (~ 0.3 S/m), and as a result, the liver would be expected to be visualized as well^22^. By overlaying relevant thoracic organs in their appropriate locations, the conductivity-based heatmaps can be better interpreted and understood (Fig. 6C). It is then clear that more conductive organs and vasculature, in addition to conductive body components located more superficially and thus closer to the sensor, generate more eddy currents and visibly reduce resistance values on the heatmap, thus confirming our hypothesis.

## Discussion

Our characterization of a continuous wearable EC pulmonary function sensor suggests translational feasibility and utility for high-risk patients or pulmonary conditions. In a series of first-in-man experiments using a static sensor, we demonstrate statistically significant linear correlations between sensor output and critical pulmonary metrics, including RR, FEV_1_, and FVC. Changing to a dynamic scanning mode allowed for point-by-point conductivity measurements and heatmap-facilitated rudimentary imaging of the thoracic cavity, which can be further honed to provide near real-time imaging of the thoracic cavity in the field setting. FEM analysis confirmed our hypothesized magnetic field distributions and sensor behavior near conductive targets. Device safety was further confirmed as the sensor operated at 1 MHz and generated magnetic fields of microtesla magnitudes.

The unique electromagnetic properties associated with EC have resulted in several precedent devices, primarily used for industrial applications. EC automobile braking systems have been developed that utilize the counteracting magnetic fields generated within the conductive target to slow vehicles. Similarly, the reliability of the EC phenomenon has also seen its use in automobile suspension systems, roller coaster braking, and elevator braking^23–26^, and nondestructive testing (NDT) has historically been utilized in the inspection and diagnosis of cracks and corrosion in the aerospace industry^27^. However, the EC phenomenon has not yet been fully investigated within the context of biomedical research. Our investigation suggests that EC sensors may accurately track changes in local conductivity, resulting from accumulation or removal of biological fluids or tissues. In addition, one major advantage of the sensor circuit is the low power consumption, which is of great importance for wearable sensors. In all trials, the sensor was powered via USB connection with a laptop, with a maximum current of 0.0027 amperes. On the same note, EC sensors demonstrate an astounding price-to-accuracy tradeoff, and the sensor utilized in this study cost less than $30 to construct, with a majority of the cost consumed by the commercial inductance-to-digital converter.

Recent work has described both the linear and non-linear dynamics between bioimpedance and respiratory metrics, such as RR, with the general consensus being that the two are linearly correlated^28–33^. This attitude within the general pulmonary literature allowed for accurate model selection, as seen in the linear regression models shown as Fig. 5. In fact, linear modeling demonstrated a statistically significant relationship between sensor output and pulmonary metrics, further supporting the nature of this relationship.

With regard to sensor construction, previous studies have investigated a wide range of sensors utilizing a vast array of physical phenomenon to predict pulmonary function. Specifically, acoustic biosensors have been developed for pulmonary diagnosis, RR, and wheeze detection^10,34,35^, mechanical biosensors have been designed to measure strain during respiration^12^, and electrical impedance tomography (EIT) has been shown to be an accurate method for non-invasive cardiopulmonary investigation^36,37^. While each methodology boasts its own unique advantages and disadvantages, the sensor developed in this study is the first reported pulmonary EC sensor in the literature. Calibration was also not a major issue for the EC sensor because it was able to predict respiratory metrics using the regression lines rather than requiring calibration datapoints for each study participant, which are still required when using strain-based biosensors^12^. In addition, the sensor architecture described in this study allows for cheap, real-time, non-contact, wearable, continuous, and accurate biosensing and crude imaging that can easily interface with electronic health records or with a physician through telehealth services.

Of particular timely concern is COVID-19 and the wide range of presentations of ARDS following infection with the virus. Although PFTs are often not performed in acutely ill patients, chronic pulmonary sequalae following COVID-19 ARDS may be accurately tracked with such a sensor. In addition, management of patients with chronic COVID-19 cannot rely on PFTs due to the high risk of respiratory droplet transmission of virus following forced expiration during spirometry^38^. As such, clinical understanding of PFTs in patients diagnosed with chronic COVID-19 sequelae continues to be limited, and non-contact wearable sensors may be able to provide additional patient-specific information regarding disease progression, and continuous sensing may hold information regarding reduced pulmonary functioning prior to clinical manifestations. To further minimize patient contact, these devices can be placed externally on the patients’ clothing or interwoven within the shirt fabric, with Bluetooth transmission to a local computer for storage and analysis.

Furthermore, since March 2020, COVID-19 has reduced the number of inpatient and elective outpatient procedures in hospitals worldwide in an effort to limit disease spread^39^. Novel biosensors capable of monitoring pulmonary function accurately may present a distinct benefit during these times. Additional uses for lung transplant patients who require frequent PFTs and diagnosis of traumatic thoracic injury such as, hemothorax, pneumothorax, or hemidiaphragm may be possible in the field setting in the future with additional development of the EC device. For all non-traumatic indications, continuous recording and digital interfacing provided by the sensor allow for remote PFT monitoring so that chronically ill patients can stay at home, thus minimizing potential COVID-19 exposure by reducing the frequency of hospital visits.

Limitations of this work include the requirement for the EC sensor to be isolated from metallic objects and magnetic fields when scanning and a small series of participants. However, the number of volunteers recruited in this study was subject to Institutional limitations stemming from the COVID-19 pandemic to prevent contact-based disease transmission. To accommodate, three participants were scanned twice. Furthermore, the resolution of heatmap-based images produced by the sensor is currently low and approximate due to the resolution of data output possible with the commercial sensor. Despite these limitations, our data shows robust evidence of the capacity of such a sensor for point-of-care pulmonary monitoring. Future work with a larger patient series aims at improving the circuit signal-to-noise ratio to improve sensor accuracy and boost the resolution of dynamic heatmap images produced through scanning. By doing so, such a device may provide information regarding critical lung metrics in sick and high-risk patients.

## Supplementary Information

Supplementary Information.

## Data Availability

The datasets generated during and/or analyzed during the current study are available from the corresponding author on reasonable request.
