# Supplementary material for: A wearable eddy current based pulmonary function sensor for continuous non-contact point-of-care monitoring during the COVID-19 pandemic
Source: Sci Rep. 2021 Oct 11;11:20144. doi: 10.1038/s41598-021-99682-2 (PMC8505507; doi:10.1038/s41598-021-99682-2)
Supplement: Supplementary file 1 — Supplementary Information. [file 41598_2021_99682_MOESM1_ESM.docx]

**A Wearable Eddy Current Based Pulmonary Function Sensor for Continuous Non-Contact Point-Of-Care Monitoring During the COVID-19 Pandemic**

Shane Shahrestani PhD, MS^1,3^, Tzu-Chieh Chou MS^2^, Kuang-Ming Shang MS^1^, Gabriel Zada MD, MS^3^, Zea Borok MD^4^, Adupa P. Rao MD^4^, Yu-Chong Tai PhD^1,2^

^1^ Department of Medical Engineering, California Institute of Technology, Pasadena, California, USA.

^2^ Department of Electrical Engineering, California Institute of Technology, Pasadena, California, USA.

^3^ Department of Neurosurgery, Keck School of Medicine, University of Southern California, Los Angeles, California, USA.

^4^ Division of Pulmonary, Critical Care and Sleep Medicine, Department of Medicine, Keck School of Medicine, University of Southern California, Los Angeles, California, USA.

**Corresponding author:**

Shane Shahrestani, PhD, MS
California Institute of Technology

Department of Medical Engineering
1200 E California Blvd, MC 136-93
Pasadena, CA 91125

+1 (714) 488-1008

sshahres@caltech.edu

**Disclosures**

This study was funded by the Merkin COVID-19 Challenge Grant. Institutional Review Board approval was obtained prior to data collection. All patient identifiers have been removed, and measures were taken to keep data de-identified and ensure privacy throughout the course of the study. There are no conflicts of interest in this study.

**Acknowledgements**

Parts of Figures 1, 2, and 6, and Supplementary Figures 1, 2, and 3 were created using Biorender.com.

**Supplemental Figures**


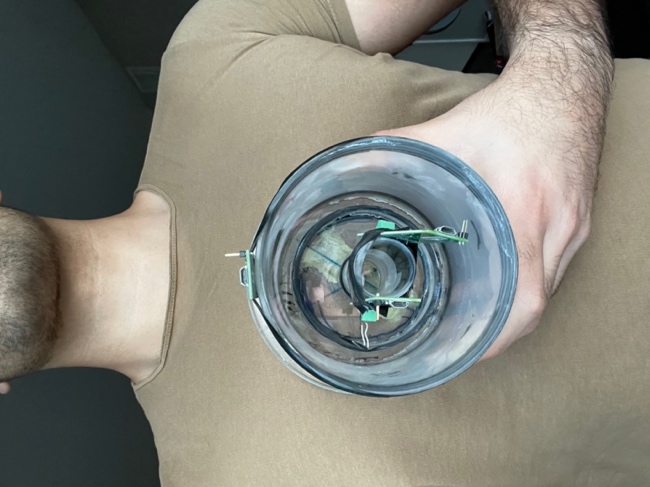

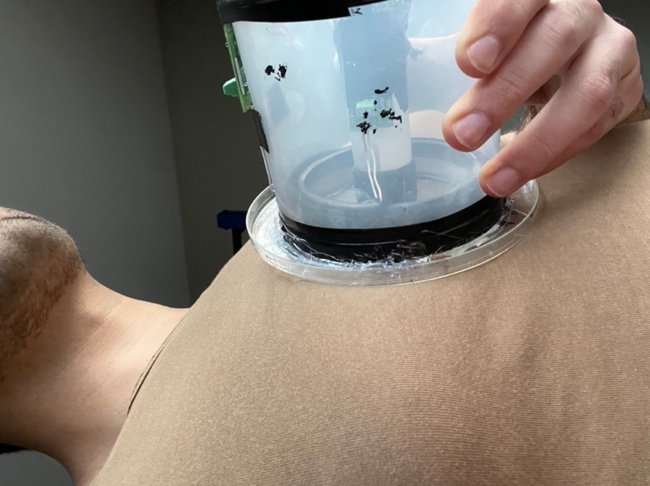

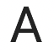

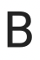

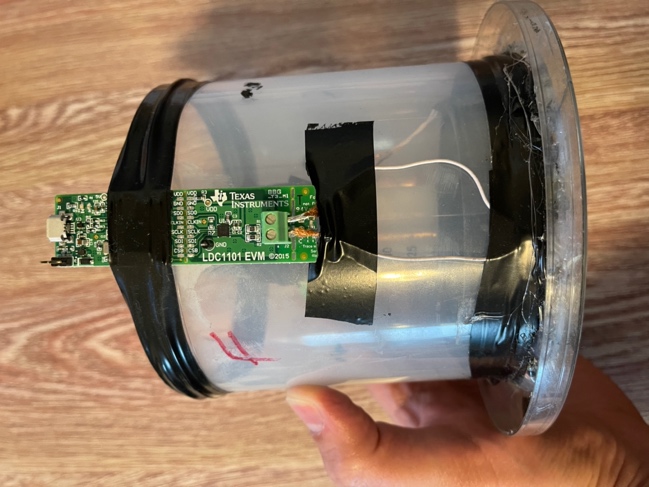

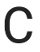


**11.4cm**

**Center of Coil at Mid-Sternum**

**USB Connection to Computer**

**Connection to LDC1101 Chip**

**Solenoid Coil with Ferrite Shielding**


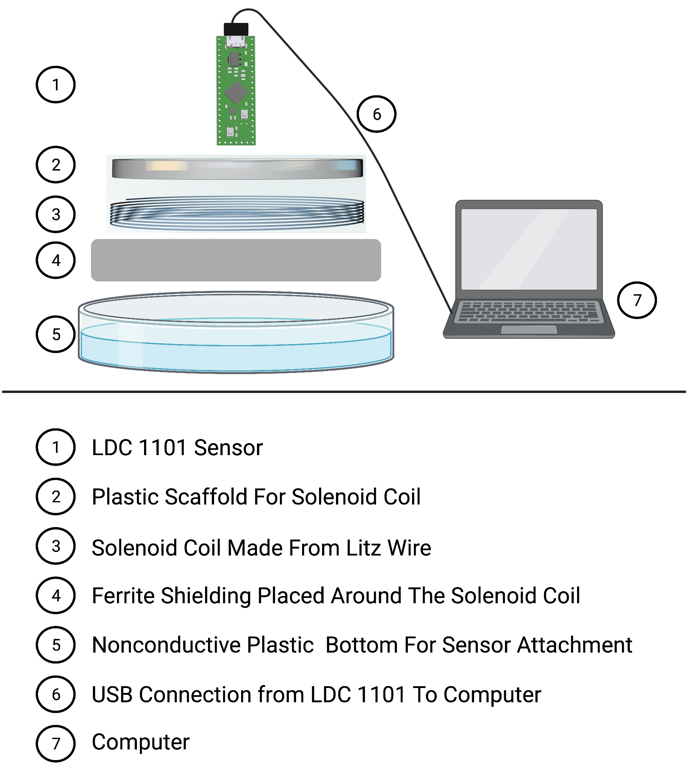

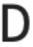


**Supplementary Fig. 1: Sensor Components and Placement. A**, Eddy current sensor placed on the participant’s shirt. Several concentric coils were developed for experimentation, but only the largest coil (11.4cm diameter) was used for all sensor output in this study. **B**, The sensor was placed such that the center of the coil was aligned with the mid-sternum of the participant. Data was recorded while the patient took breaths. **C**, The sensor includes an outer solenoid ring of Litz wire with ferrite shielding (can’t be visualized because it was taped to the coil). The wire was connected to the LDC1101 chip, which was then connected via USB to a computer for digital readout. **D**, Sensor components shown in a deconstructed sensor. All relevant parts are numerically coded.

**Supplementary Fig. 2: Unfiltered respiratory waveform output. A-J**, Each waveform shows the unique unfiltered pulmonary waveform, as generated by the sensor, for each trial in this study. Savitzky-Golay and simple Gaussian filters from the MATLAB Signal Processing Toolbox were implemented to smooth the respiratory time series waveforms and to remove high frequency noise. After filtering (Fig. 3 in manuscript), local maxima and minima were located within each respiratory diagram and the difference between the peaks and troughs were averaged for each individual trial for all breaths, yielding the average change in R_p_ per breath. An example of this process is shown in subfigure J for explanation, although the actual calculations were performed with the filtered waveforms in Fig. 3.


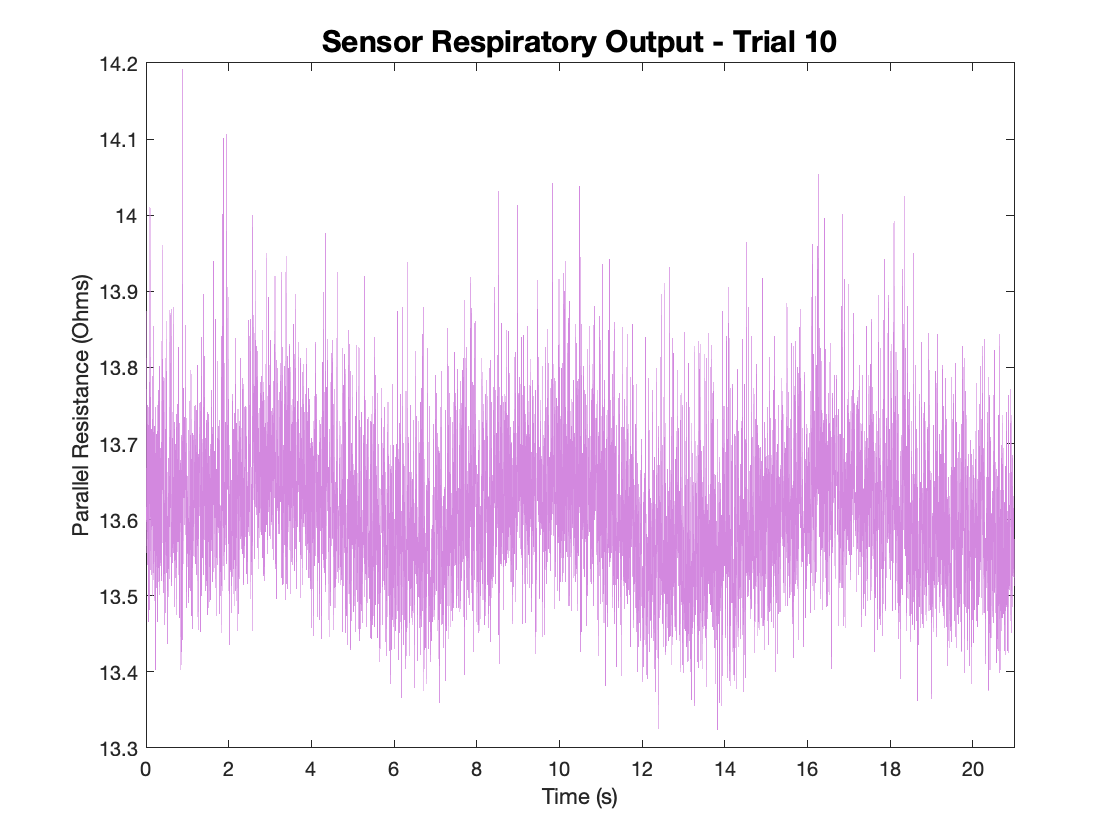

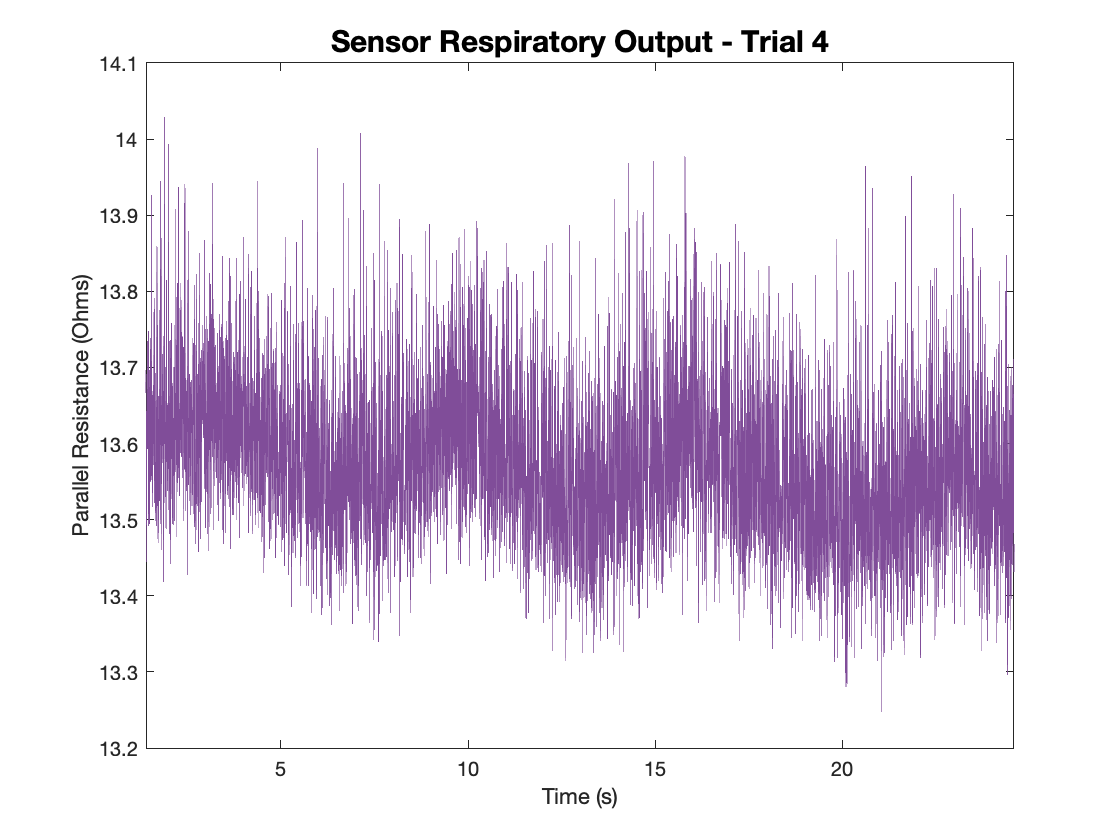

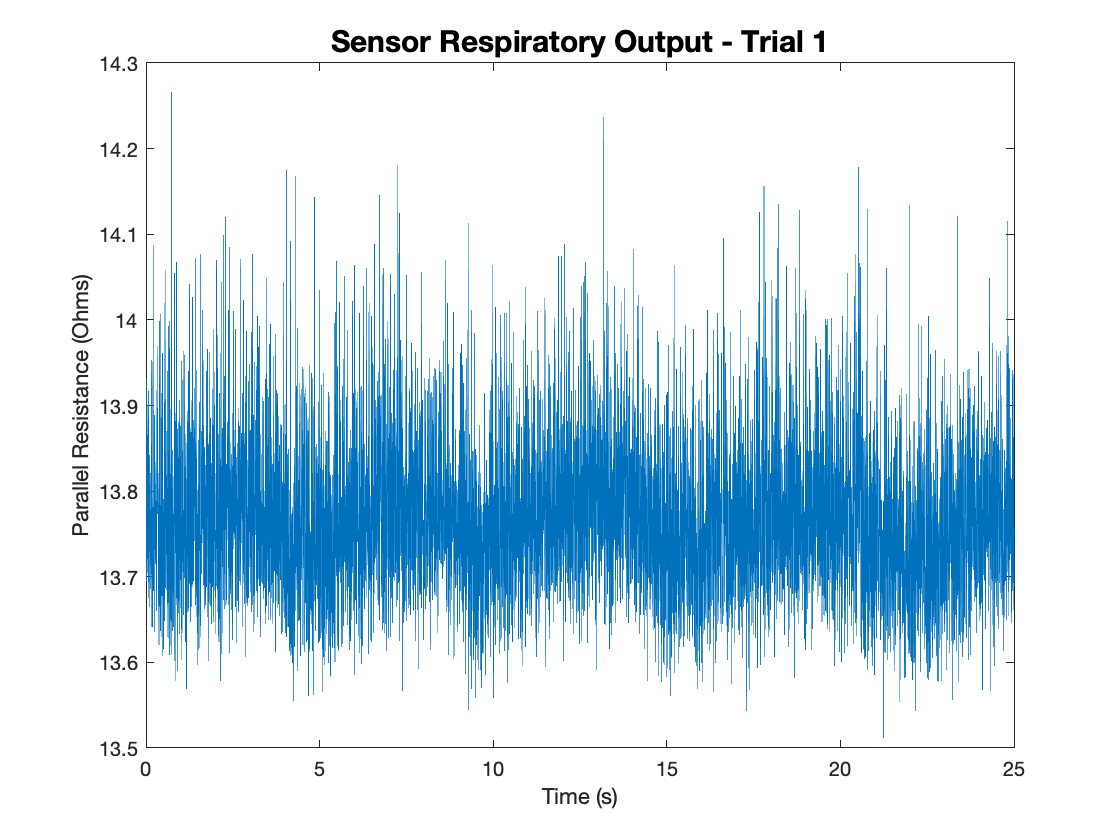

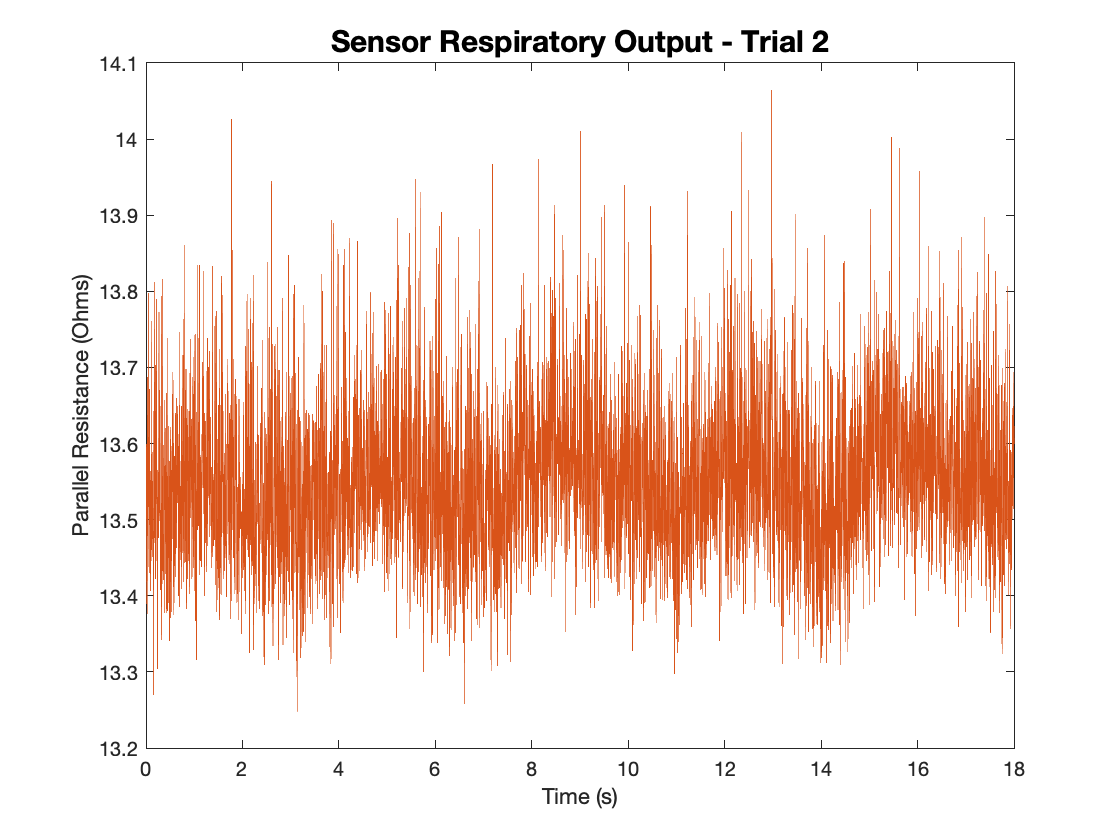

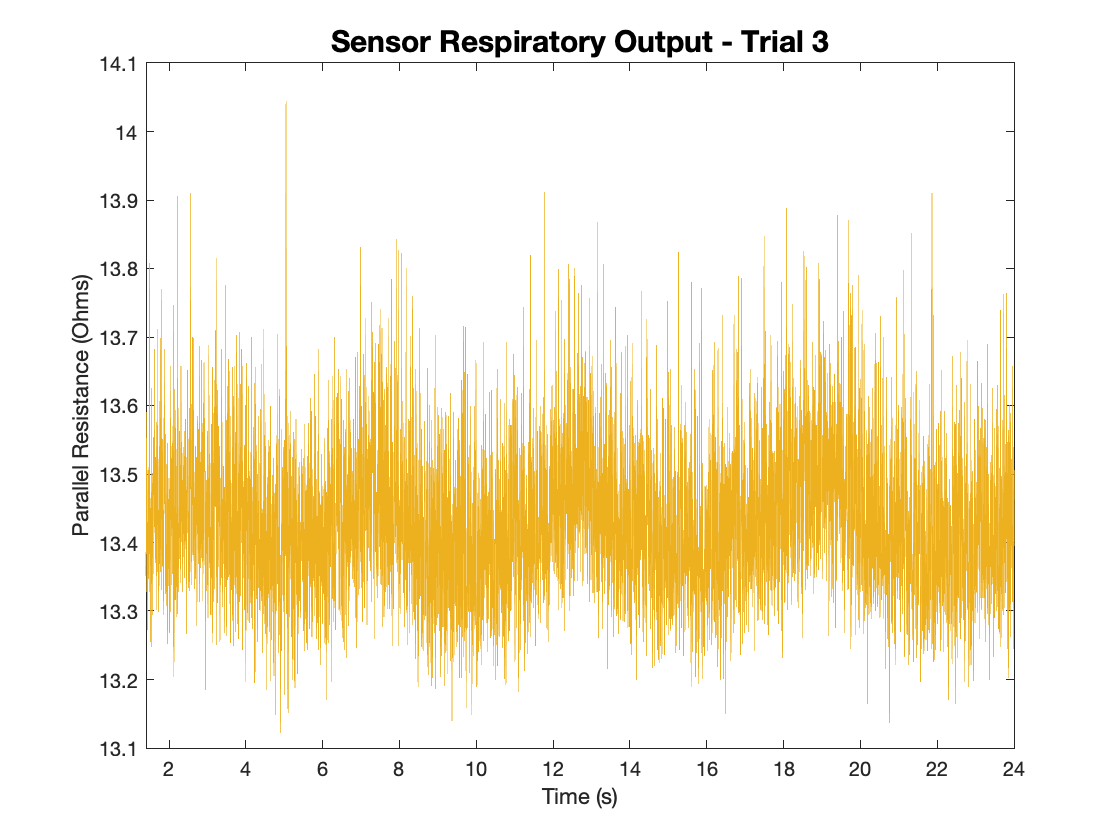

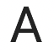

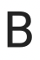

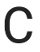

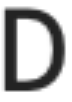

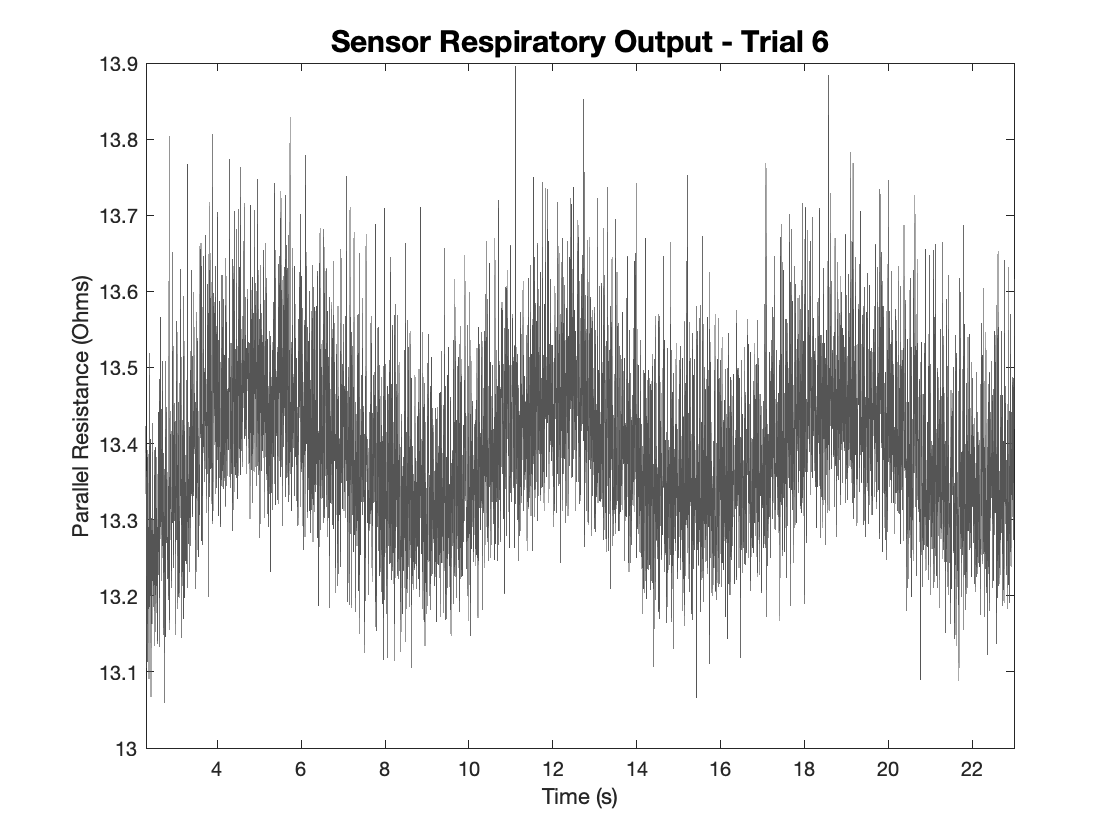

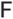

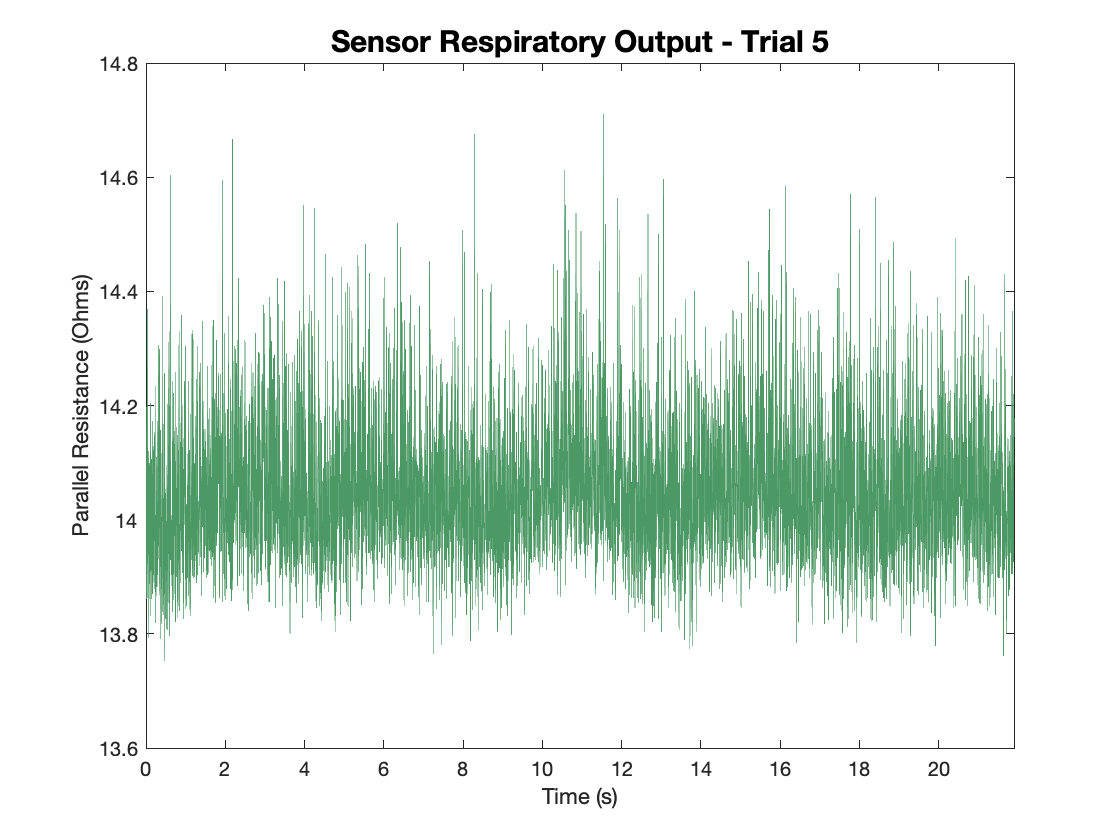

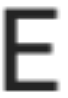

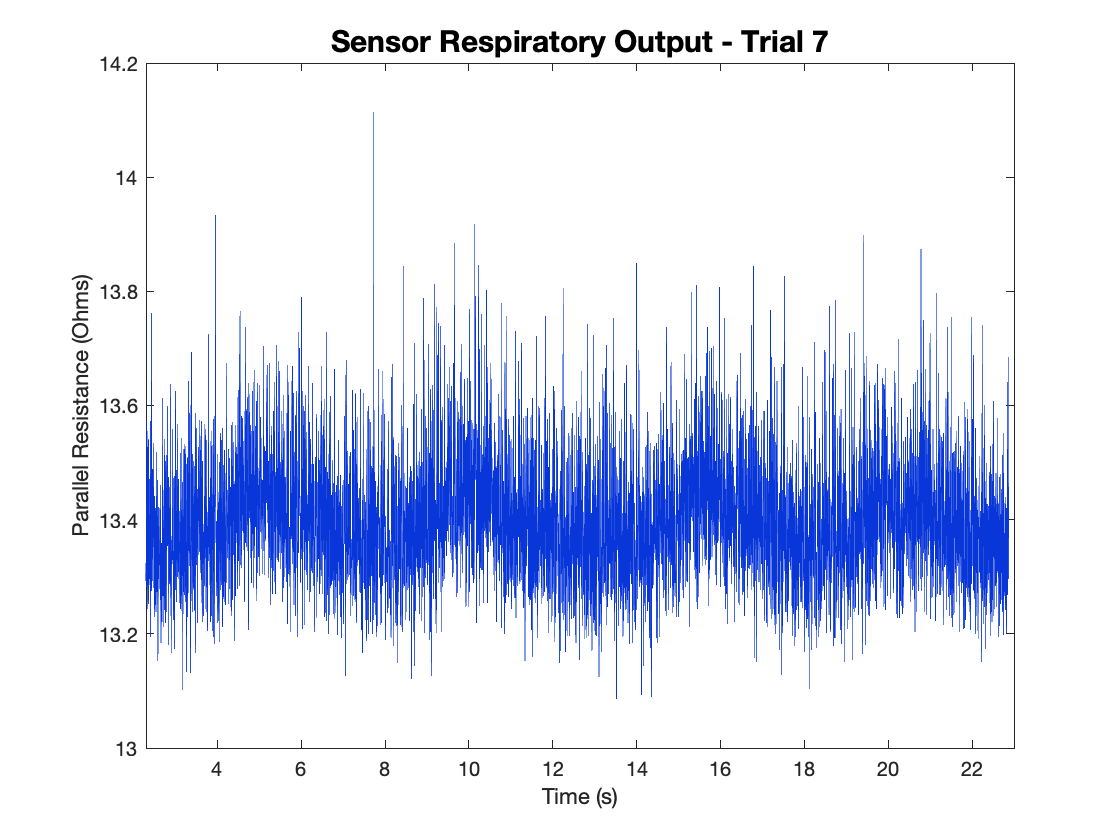

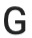

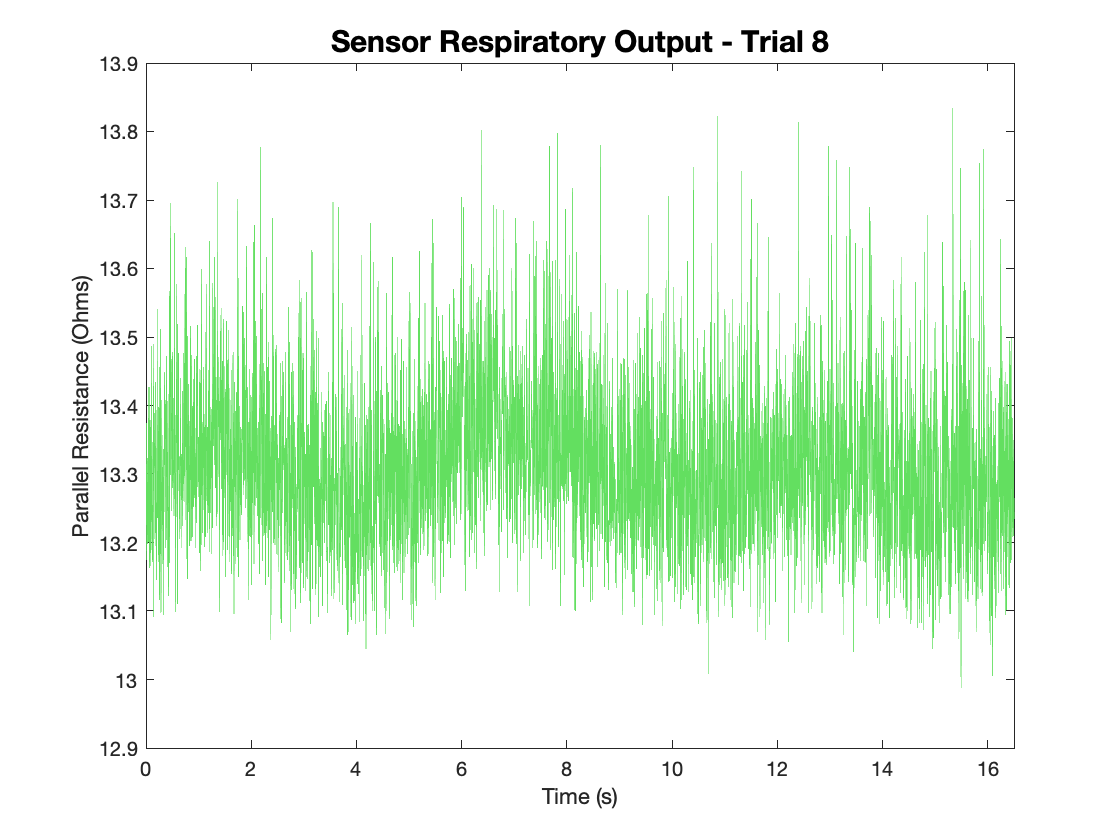

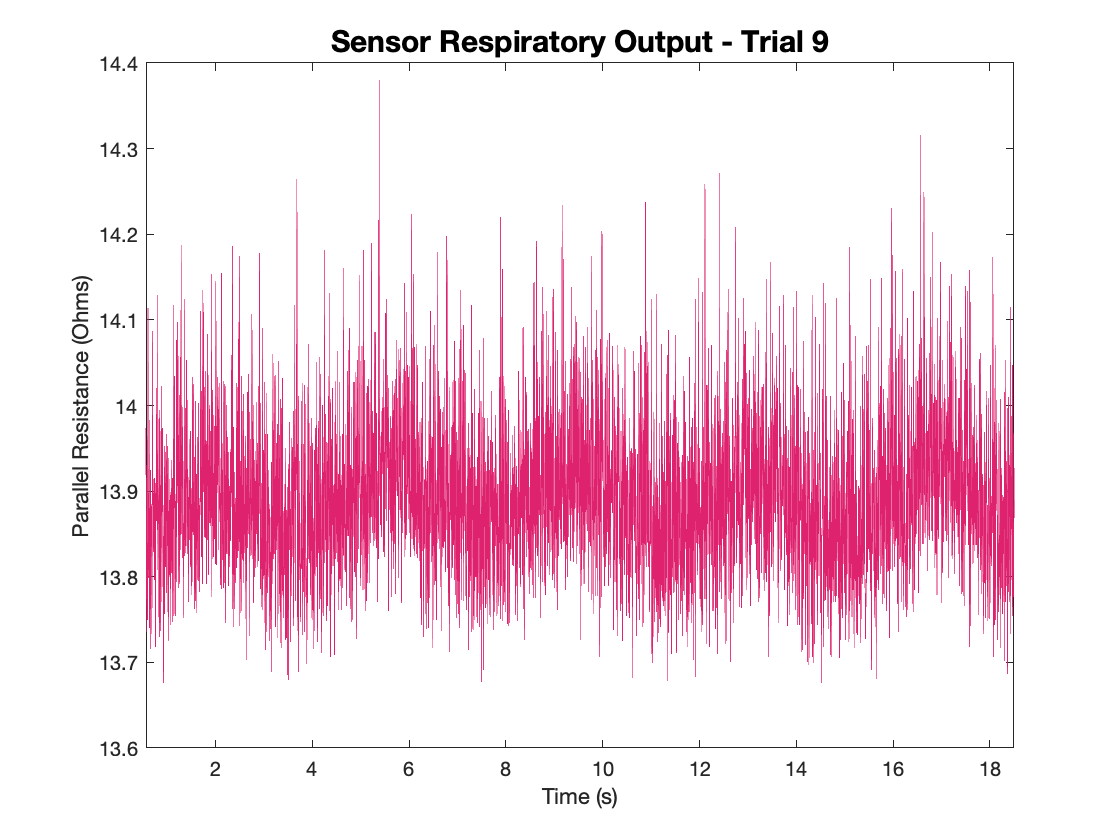

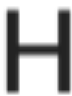

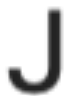

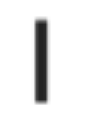


**Local Maximum**

**Local Minimum**

$\boldsymbol{\Delta}$**R_p_^1^**

$\boldsymbol{\Delta}$**R_p_^2^**

$\boldsymbol{\Delta}$**R_p_^3^**

$\boldsymbol{\Delta}$**R_p_** = ($\boldsymbol{\Delta}$**R_p_^1^+** $\boldsymbol{\Delta}$**R_p_^2^+** $\boldsymbol{\Delta}$**R_p_^3^)/3**


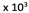

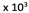

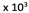

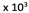

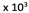

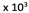

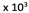

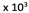

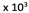

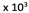


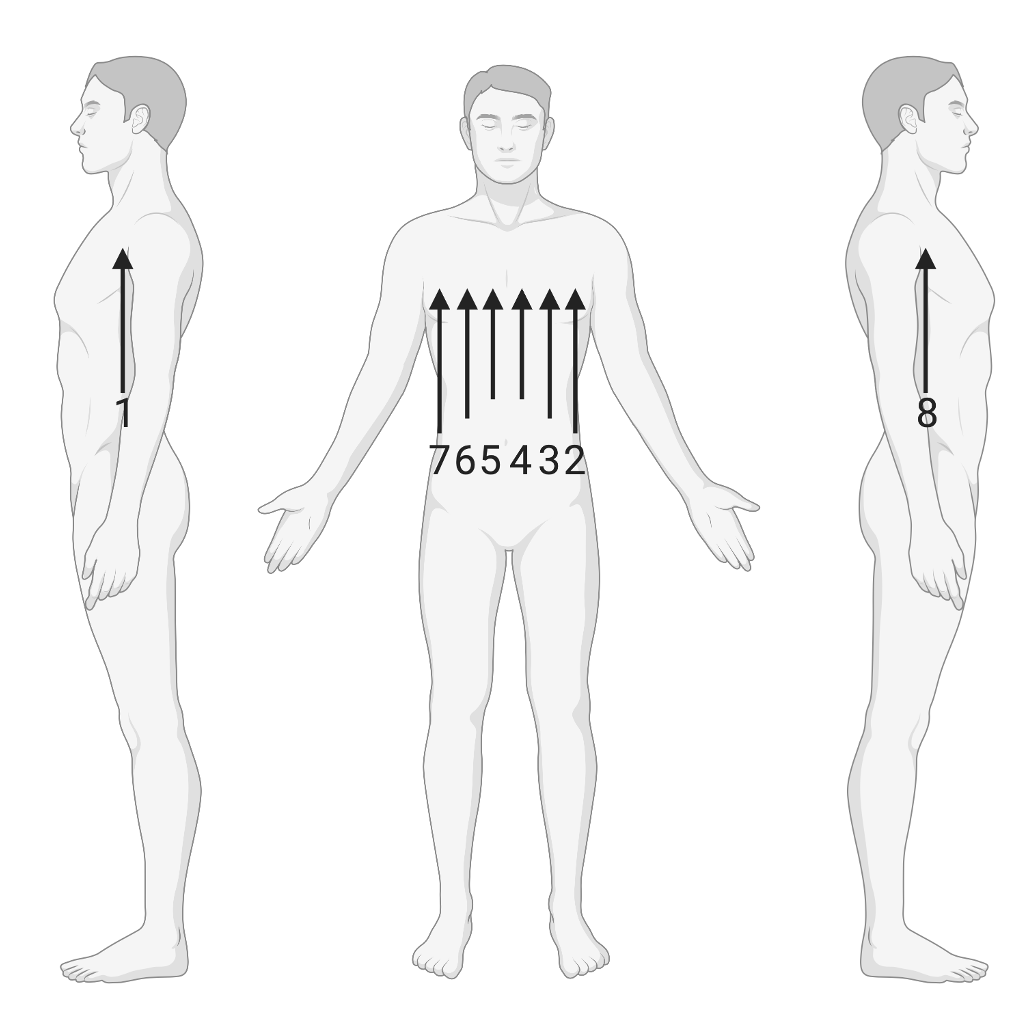
**Supplementary Fig. 3: Dynamic Scanning Path.** Scanning the anterior chest wall started at the left mid-axillary line (row 1) and continued to the right mid-axillary line (row 8), vertically from the inferior border of the ribs to the clavicles superiorly. Data was saved in real-time for heatmap production.

ECD vs Stray Capacitance Analysis

The LC tank with parallel resistance is shown as Supplementary Fig. 5A. Here, we demonstrate the LC tank circuit spectrum under three conditions: without any ferromagnetic/conductive materials near the LC tank (Supplementary Fig. 5B), LC tank placed on the chest when exhaling (Supplementary Fig. 5C), and LC tank placed on the chest when inhaling (Supplementary Fig. 5D). Comparing the results with and without sensor on the chest, it is shown that the resonant frequency does not change, meaning the stray capacitance coupled from the coil to the human tissue does not dominate. Meanwhile, the main difference between LC tank on the chest and not on the chest is the decrease in Rp at the resonant frequency, which originates from the energy loss caused by the eddy currents generated in the conductive human tissue. Analyzing the degree of decrease in Rp when exhaling or inhaling will provide us detailed information regarding the air volume change in the lung.

| **A.**  **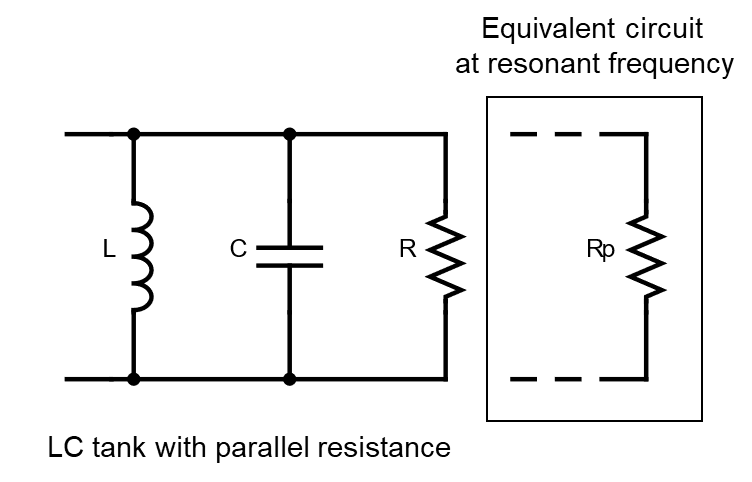** | **B. with no ferromagnetic/conductive material nearby**  **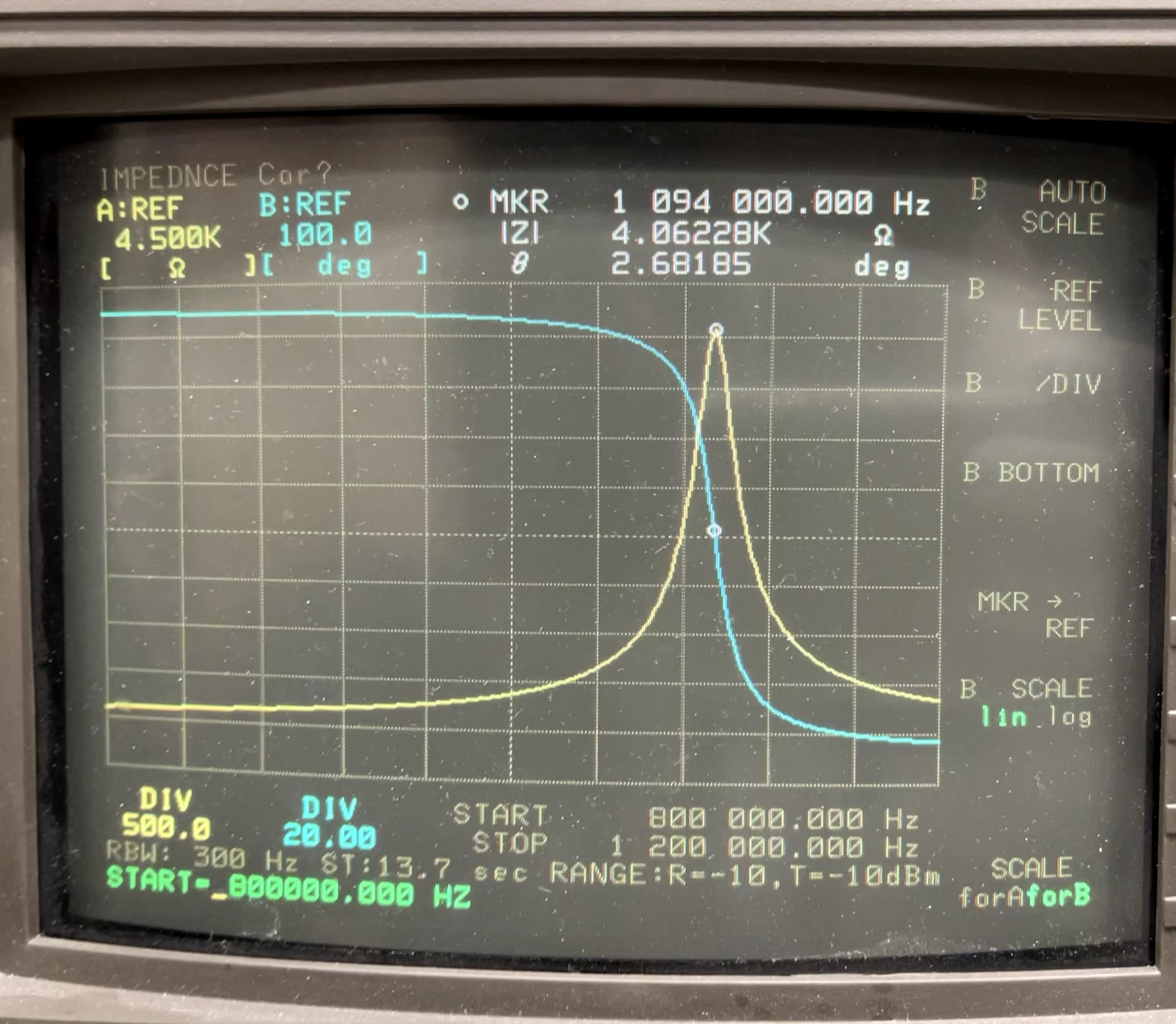** |
| --- | --- |
|  | **Resonant frequency = 1.094 MHz**  **Rp = 4.062 kOhm** |
| **C. on the chest when exhaling**  **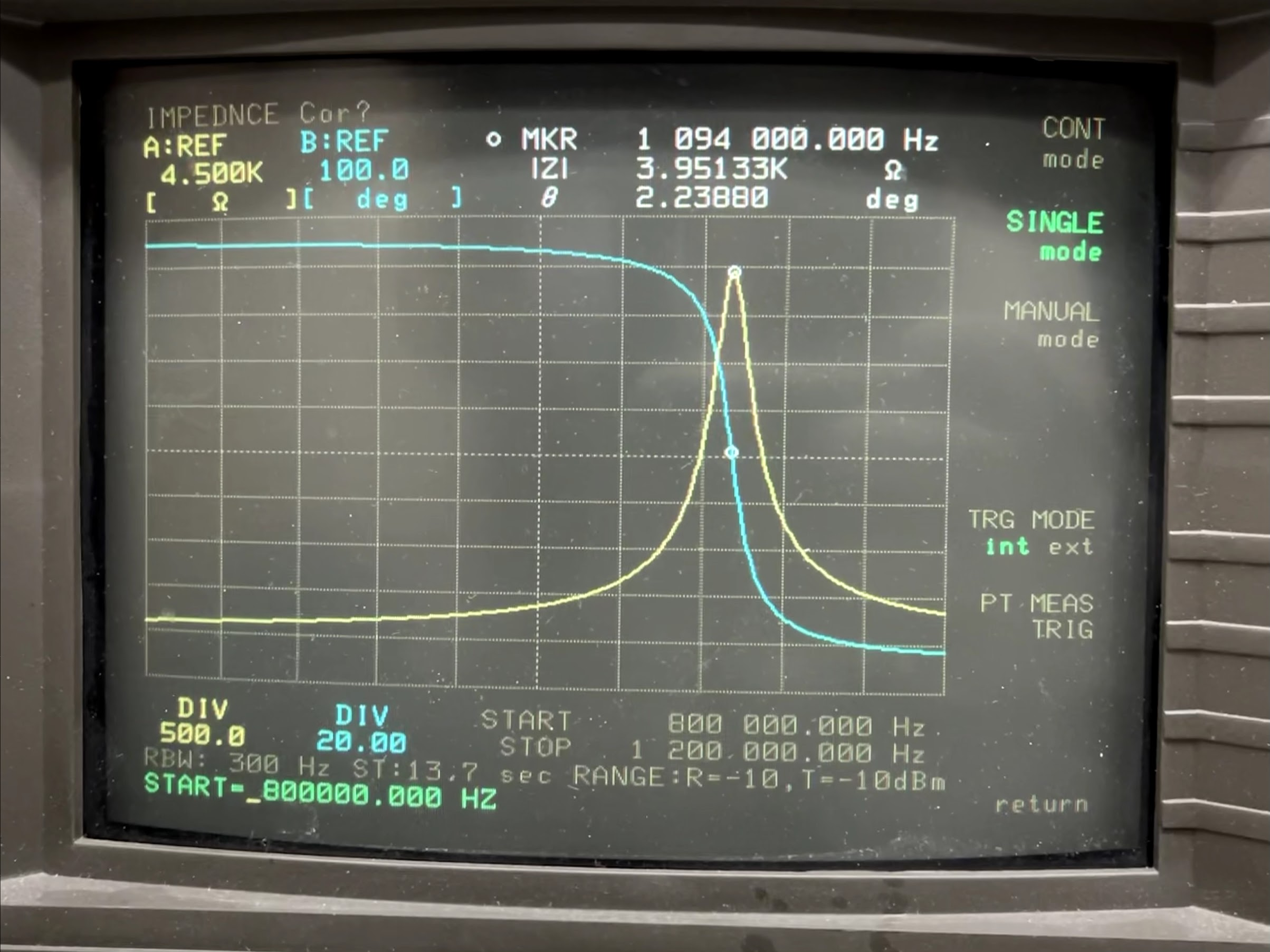** | **D. on the chest when inhaling**  **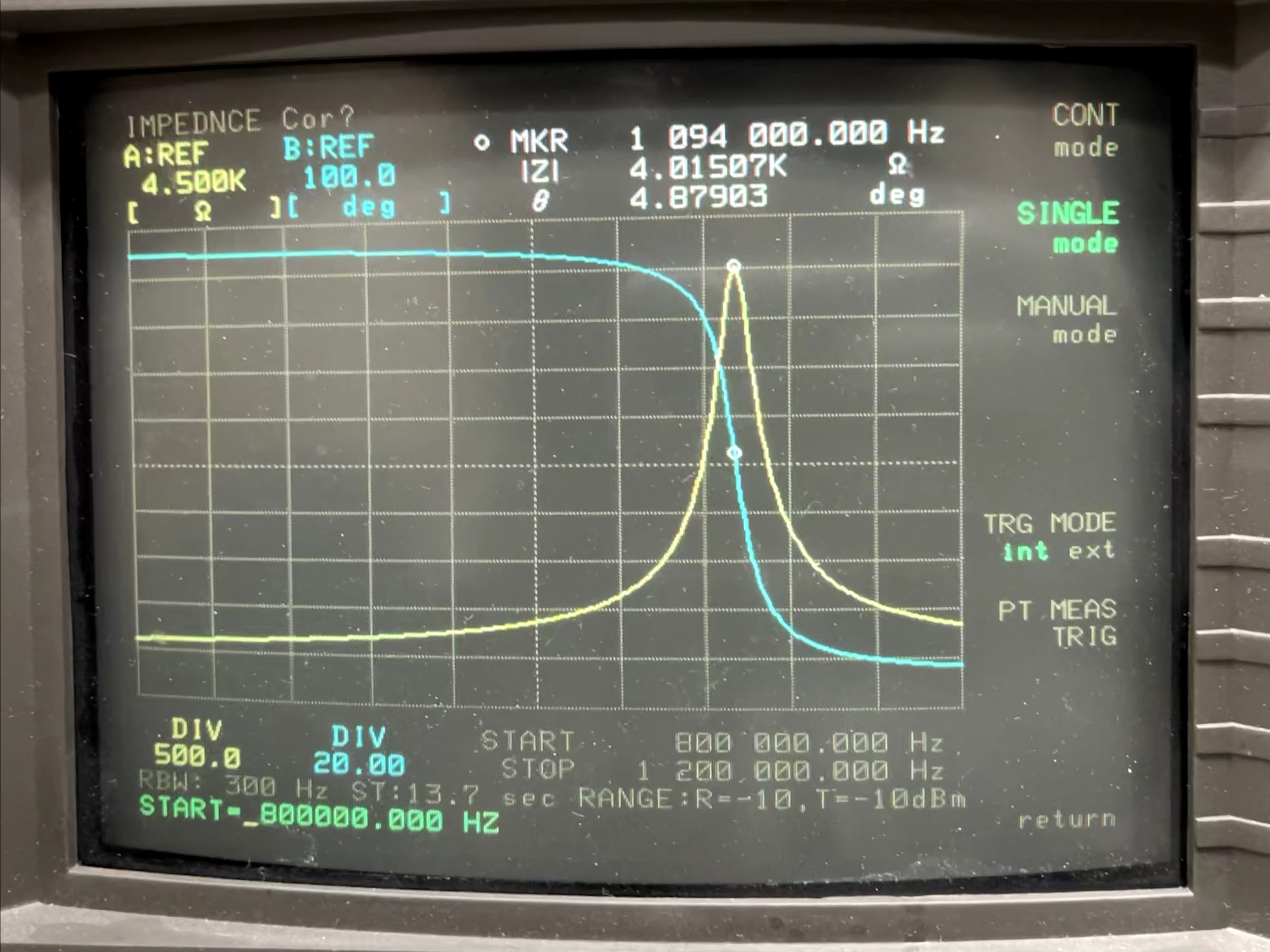** |
| **Resonant frequency = 1.094 MHz**  **Rp = 3.951 kOhm** | **Resonant frequency = 1.094 MHz**  **Rp = 4.015 kOhm** |
| **Supplementary Fig. 4:** LC tank circuit (A) used in the eddy current sensor and the LC tank impedance measurement results (B) with no ferromagnetic/conductive material nearby; (C) on the chest when exhaling; and (D) on the chest when inhaling. The peak value in the amplitude (\|Z\|, yellow line) is equal to Rp at the resonant frequency. | |
